# Association Between Maternal Continuum of Care Utilization and Childhood Undernutrition in Bangladesh: Findings from Nationally Representative Surveys

**DOI:** 10.3390/nu18121847

**Published:** 2026-06-08

**Authors:** M. A. Rifat, Sanjib Saha

**Affiliations:** 1 BRAC Institute of Educational Development, BRAC University, Dhaka 1212, Bangladesh; rifatahmed011@gmail.com; 2 Department of Clinical Science (Malmö), Lund University, 22362 Lund, Sweden

**Keywords:** undernutrition, children, maternal care, ANC, PNC, BDHS, Bangladesh

## Abstract

Background/Objectives: Antenatal Care (ANC), delivery assisted by Skilled Birth Attendants (SBA), and Postnatal Care (PNC), collectively referred to as Continuum of Care (CoC), are platforms for delivering maternal and child nutrition interventions essential for optimal child growth. The study objective is to estimate the association between maternal utilization of CoC and childhood undernutrition in Bangladesh. Methods: Data were obtained from two nationally representative cross-sectional surveys, including Bangladesh Demographic and Health Survey (BDHS) 2017–18 and BDHS 2022. Childhood undernutrition was assessed using five indicators: (1) stunting, (2) wasting, (3) underweight, (4) any undernutrition (presence of any of first three conditions), and (5) multiple undernutrition (presence of at least two of first three conditions). Multivariable logistic regression models were used to estimate survey-specific and pooled associations. Results: Maternal utilization of full CoC was significantly associated with 51%, 35%, and 48% lower odds of childhood wasting, underweight, and multiple undernutrition in the BDHS 2022 sample, respectively. Receiving a PNC within 48 hours of delivery was significantly associated with 15% lower odds of stunting and any undernutrition in the BDHS 2017–18 sample and 17% lower odds of stunting and any undernutrition in the pooled sample. No significant differences in the survey-specific effects of maternal utilization of full CoC and its components on childhood undernutrition were observed; however, relatively stronger protective effects were evident in BDHS 2022 compared to those in BDHS 2017–18. The association between maternal utilization of full CoC and childhood stunting was not significant. Conclusions: The effectiveness of maternal utilization of CoC in preventing acute and concurrent forms of childhood undernutrition in Bangladesh has improved over time. However, substantial gap exists in translating these benefits to a reduction in chronic childhood undernutrition.

## 1. Introduction

Childhood undernutrition stands as one of the major public health problems globally, cascading both short-term and long-term consequences at individual, family and societal levels [1,2]. Short-term consequences of child undernutrition encompass elevated risk of morbidity, mortality and disease burden, which are primarily due to disrupted immune function as a result of inadequate nutrient supply in the body [3,4,5]. On the other hand, long-term consequences of child undernutrition include reduced human potential, poor cognition, permanent growth failure, and low economic productivity, with the possibility of bringing these consequences to the next generations [1,6]. Reasonably, height-for-age until two years of age is mentioned as the best predictor of human capital [1]. Although global childhood undernutrition has been reduced significantly over decades, it is still a major concern in low-income and middle-income countries [7]. In Bangladesh, the prevalences of stunting, wasting, and underweight among children under five years of age were 31%, 8%, and 21%, respectively [8]. Furthermore, one out of five children were suffering from multiple forms of undernutrition, i.e., the presence of at least two out of stunting, wasting, and underweight [9].

To prevent childhood undernutrition in Bangladesh, one of the major strategies adopted by National Nutrition Policy 2015 and Second National Plan of Action for Nutrition (2016–2025) emphasized on integrating nutrition services into existing maternal, neonatal and child health (MNCH) service platforms, e.g., nutrition intensified maternal care from prenatal to postnatal periods [10,11]. Continuum of Care (CoC), a major component of MNCH services, offers a comprehensive package of services required for improved health, well-being, and survival of mothers and children in critical stages, e.g., pregnancy, delivery, and the postnatal period [12,13,14]. Furthermore, CoC is a crucial opportunity for sensitizing mothers about care practices and delivering essential nutrition services. For example, Antenatal Care (ANC) is a platform for providing nutrition counseling and essential micronutrient supplements, e.g., iron, folic acid, and calcium, essential for ensuring adequate nutrient supply required for intrauterine growth and maternal nourishment [10]. Care during delivery, e.g., early mother–child skin-to-skin contact and initiation of breastfeeding, was found to contribute long-term health and nutritional benefits in mothers and infants [15]. A Postnatal Care (PNC) visit within 48 hours of delivery provides an extra layer of protection for mothers and newborns who need further healthcare support. PNC is also an opportunity to inform mothers about infant feeding practices, e.g., exclusive breastfeeding [10].

Following the adoption of the National Nutrition Policy in 2015, the delivery of maternal nutrition interventions through CoC platforms for maternal health has been strengthened in Bangladesh [10]. However, previous studies have identified both opportunities and challenges in the integration of health and nutrition service delivery, in relation to service quality, logistical availability, and capacity of service providers, leaving it inconclusive whether there was a significant reflection of CoC for maternal health on child nutrition [16,17]. Moreover, the magnitude of association between maternal utilization of CoC and nutritional status of children under five years of age in Bangladesh remains unexamined. This study was conducted to address this knowledge gap. Publicly available data from Bangladesh Demographic and Health Surveys (BDHS) conducted after 2015 were utilized for the analyses. Findings can be useful for policymakers in decision-making process, including design and implementation of interventions, priority setting, and the review of existing policies.

## 2. Materials and Methods

### 2.1. Study Design, Data Sources and Sample

The study is cross-sectional. Bangladesh Demographic and Health Survey (BDHS) 2017–18 and BDHS 2022 were utilized as the data sources [8,18]. This survey is nationally representative and employed two-stage stratified cluster random sampling. The methodology of the surveys and variable descriptions are detailed elsewhere [8,18,19,20]. The overall response rate of the surveys was more than 98%. In this study, Strengthening the Reporting of Observational studies in Epidemiology (STROBE) was considered as the reporting guideline (Supplementary Material Table S1) [21].

**Figure 1:**
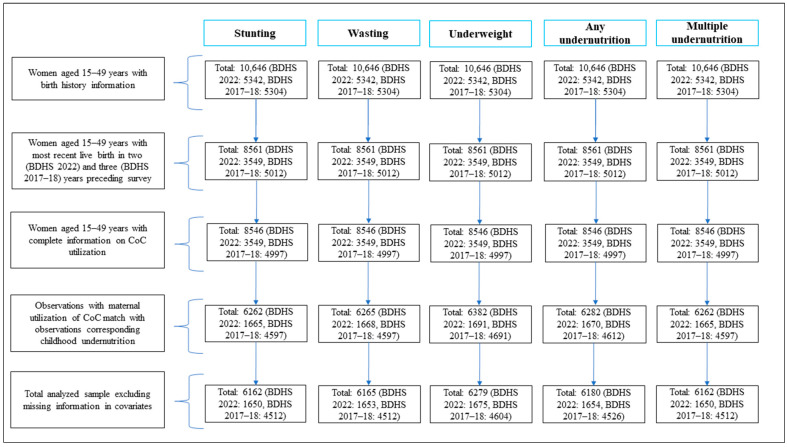
Selection process of the analyzed sample. Note: BDHS (Bangladesh Demographic and Health Survey); CoC (Continuum of Care).

To obtain the analyzed sample, observations corresponding women aged 15–49 years who had the most recent live birth within the last three years and two years preceding the surveys were isolated from BDHS 2017–18 and BDHS 2022 data, respectively [8,18]. Consideration of different time frames for these two surveys was based on the survey guidelines. The most recent live birth was considered so that it includes children whose nutritional status was possible to estimate. These observations were considered as the denominator to estimate maternal utilization of CoC. Finally, observations corresponding maternal utilization of CoC information were matched with observations containing childhood undernutrition information. The analyzed sample included observation with no missing information in maternal utilization of CoC, childhood undernutrition, and covariates. As the information about maternal CoC was available for mothers who had the most recent live birth within three (BDHS 2017–18) and two (BDHS 2022) years preceding the surveys, the analyzed sample did not include all children under five years of age but those who corresponded to the maternal CoC information. The analyzed samples represent children of corresponding age groups in Bangladesh given that they cover all the divisions and representative sampling strata (rural, city corporations, and urban areas other than city corporations). The selection process is illustrated in Figure 1.

### 2.2. Exposure Variables

The exposure variable was maternal utilization of CoC, which consists of three integral components essential for comprehensive maternal healthcare spanning from pregnancy through the post-delivery period. Although there are country-specific standards, in the Bangladesh context, these three components include (1) at least four ANC by skilled healthcare providers during pregnancy, (2) delivery assisted by SBA, and (3) at least one PNC by skilled healthcare providers within 48 hours of delivery [22,23]. Here, skilled healthcare providers refer to doctors, nurses, midwives or paramedics, family welfare visitors, community Skilled Birth Attendants, and sub-assistant community medical officers. A mother was considered to receive CoC if she utilized all three components. While preparing analyzed sample, the positive status of maternal utilization of full CoC was coded as 1; otherwise, it was 0.

### 2.3. Outcome Variables

The status of childhood undernutrition categories represented the outcome variable. These include (1) stunting (height-for-age Z-score < −2 SD), (2) wasting (weight-for-height Z-score < −2 SD), (3) underweight (weight-for-age Z-score < −2 SD), (4) any undernutrition (positive status of any of stunting, wasting, and underweight), and (5) multiple undernutrition (presence of at least two of stunting, wasting, and underweight) [19]. In the survey datasets, there were some children with missing information in stunting, wasting, or underweight. Therefore, while assessing any childhood undernutrition, the status was labeled as missing if there was no information about any one of these three categories although the child was not undernourished according to the remaining two categories. Similarly, the status of multiple undernutrition was labeled as missing if any one out of stunting, wasting, and underweight was missing although a child was undernourished in one of the remaining two categories. While coding, a positive status in childhood undernutrition categories was coded as 1; otherwise, children were coded as 0.

### 2.4. Covariates

**Figure 2:**
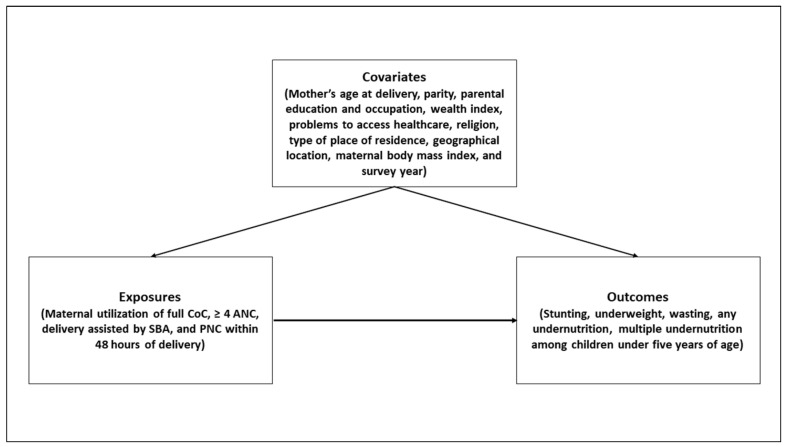
Relationship among exposures, outcomes, and covariates as demonstrated by Directed Acyclic Graph (DAG). Note: ANC (Antenatal Care); CoC (Continuum of Care); PNC (Postnatal Care); SBA (Skilled Birth Attendant).

The selection of covariates was based on their expected associations with exposures and outcomes as identified from the published literature and the use of a Directed Acyclic Graph (DAG) as demonstrated in Figure 2 [9,24,25,26]. Therefore, covariates considered for analysis were (1) mothers’ age at delivery (<19 years, 19–30 years, 31–49 years), (2) mother’s education (no education, primary, secondary, higher), (3) father’s education (no education, primary, secondary, higher), (4) parity (1, 2–3, >3), (5) mother’s occupation (not working, working), (6) father’s occupation (not working, working), (7) wealth index (poorest, poorer, middle, richer, richest), (8) whether mothers faced any problems in accessing healthcare, e.g., obtaining permission from their husband to access healthcare, getting monetary support, or distance from health facilities (big problem, not a big problem), (9) religion (Muslim, other), (10) type of place of residence (rural, urban), (11) division of residence (Barisal, Chittagong, Dhaka, Khulna, Mymensingh, Rajshahi, Rangpur, Sylhet), (12) body mass index (BMI) of mother (underweight, normal, overweight, obese), and (13) survey round (BDHS 2017–18, BDHS 2022).

### 2.5. Statistical Analysis

The distribution of childhood undernutrition categories by the status of maternal utilization of CoC and covariates was observed using cross-tabulations and the differences were observed by Chi-squared tests.

The association between childhood undernutrition categories and maternal utilization of CoC was estimated by logistic regression models, adjusted for primary sampling units, sampling strata, sampling weights, and covariates. Effect sizes were calculated to observe the associations of childhood undernutrition categories with maternal utilization of full CoC and its components, i.e., ≥4 ANC, delivery assisted by SBA, and a PNC within 48 hours of delivery. To observe survey-specific and overall effects, all the analyses were conducted in BDHS 2017–18, BDHS 2022, and pooled datasets. Equality of survey-specific associations between exposures and outcomes were observed using joint Wald tests for the interaction coefficients. As child age distributions were different across surveys (0–3 years in BDHS 2017–18 and 0–2 years in BDHS 2022), sensitivity analyses were performed using only children aged 0–2 years in both surveys.

The Variance Inflation Factor (VIF) was observed to examine the multicollinearity in the multivariable logistic regression models. A VIF < 5 was considered as an indication of multicollinearity within acceptable level [27]. All the statistical tests were conducted considering *p* < 0.05 as statistical significance. The software STATA version 17 (StataCorp, College Station, TX, USA) was used for data analysis.

## 3. Results

### 3.1. Sample Characteristics

**Figure 3:**
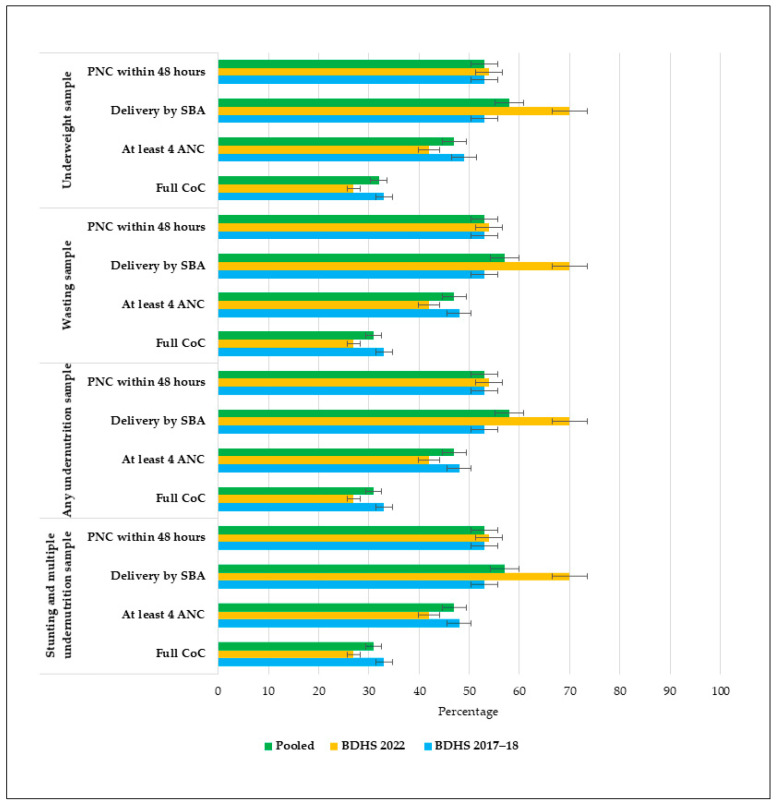
Distribution of maternal utilization of CoC and its components by childhood undernutrition categories in the analyzed sample. Note: ANC (Antenatal Care); BDHS (Bangladesh Demographic and Health Survey); CoC (Continuum of Care); PNC (Postnatal Care); SBA (Skilled Birth Attendant). Error bars represent standard error of percentages.

In the analysis, 6162, 6165, 6279, 6180, 6162 mother–child pairs were included in the pooled sample for stunting, wasting, underweight, any undernutrition, and multiple undernutrition, respectively (Table 1). In the pooled sample, the proportion of children with stunting, wasting, underweight, any undernutrition, and multiple undernutrition was 28.11%, 8.81%, 19.03%, 36.44%, 17.10%, respectively. Except underweight, the distribution of other childhood undernutrition categories differed significantly by year of survey. Considering the distribution of maternal utilization of CoC and its components in the analyzed sample, a slight variation was observed across BDHS 2017–18, BDHS 2022, and the pooled sample (Figure 3).

The proportion of mothers receiving full CoC and at least four ANCs was higher in BDHS 2017–18, whereas the proportion of mothers having delivery assisted by SBA and PNC within 48 hours of delivery was higher in the BDHS 2022 sample. In the pooled sample, the distribution of childhood stunting, underweight, any undernutrition, and multiple undernutrition differed significantly (*p* < 0.05) by the status of maternal utilization of full CoC, ≥4 ANC, delivery assisted by SBA, PNC within 48 hours of delivery, parity, mother’s education, father’s education, mothers’ problems in accessing healthcare, maternal BMI, wealth index, type of place of residence, and division (Table 1). The distribution of childhood wasting differed significantly by the status of maternal utilization of full CoC (*p* = 0.005), delivery assisted by SBA (*p* = 0.023), PNC within 48 hours of delivery (*p* = 0.005), maternal education (*p* < 0.001), maternal BMI (*p* < 0.001), and year of survey (*p* = 0.007).

### 3.2. Association Between Maternal Utilization of CoC and Childhood Undernutrition

Survey specific and overall association between childhood undernutrition categories and maternal utilization of CoC are presented in Table 2. The analysis revealed a general protective nature of maternal utilization of full CoC and its components against childhood stunting and any undernutrition in all analyzed samples. However, maternal status of receiving full CoC was significantly associated with 51% (95% CI of AOR: 0.30, 0.80, *p* = 0.01), 35% (95% CI of AOR: 0.43, 0.98, *p* = 0.04), and 48% (95% CI of AOR: 0.34, 0.81, *p* = 0.004) lower odds of childhood wasting, underweight, and multiple undernutrition in the BDHS 2022 sample. A similar significant protective nature of maternal status of receiving ≥4 ANC was observed against childhood wasting, underweight, and multiple undernutrition but in the BDHS 2022 sample only. On the other hand, maternal status of having delivery assisted by SBA was significantly associated with 16% (95% CI of AOR: 0.73, 0.97, *p* = 0.02), 15% (95% CI of AOR: 0.72, 0.998, *p* = 0.048), and 16% (95% CI of AOR: 0.73, 0.96, *p* = 0.01) lower odds of childhood stunting, underweight, and any undernutrition in the pooled sample. A similar protective nature of maternal status of receiving a PNC within 48 hours of delivery against childhood stunting, underweight, any undernutrition, and multiple undernutrition was observed in the pooled sample. In the BDHS 2017–18 sample, maternal status of receiving a PNC within 48 hours of delivery was significantly associated with reduced childhood stunting and any undernutrition. However, maternal status of receiving full CoC was not significantly associated with childhood stunting and any undernutrition in either individual surveys or pooled sample.

The joint Wald tests revealed no significance difference in associations between maternal CoC utilization and childhood undernutrition across BDHS 2017–18 and BDHS 2022 surveys. Sensitivity analyses conducted with samples including children aged 0–2 years in both surveys revealed that the pattern of association remains the same in both BDHS 2017–18 and BDHS 2022 surveys. For example, in the majority of the models, stronger protective effects of maternal utilization of CoC and its components on childhood undernutrition categories were observed in BDHS 2022. On the other hand, stronger effects of delivery assisted by SBA and a PNC within 48 hours of delivery on underweight were observed in BDHS 2017–18 in both analyses. Findings from sensitivity analysis are presented in Supplementary Material (Supplementary Material Table S2).

In the analyzed samples, fewer than 2% of observations were excluded due to missing information in covariates. We conducted analysis with observations containing missing values in covariates and compared them with the presented results. We did not find any difference in the status of significance of effect sizes between analyses conducted with and without missing values in covariates. In all the multivariable logistic regression models, VIF across covariates was <5, indicating an acceptable level of multicollinearity.

## 4. Discussion

In this study, the effects of maternal utilization of CoC and its components on childhood undernutrition categories were not consistent, with significant differences in survey-specific effects where protective effects of maternal utilization of CoC and its components on childhood undernutrition were stronger in BDHS 2022.

In BDHS 2017–2018 and the pooled sample, neither maternal utilization of full CoC nor receiving ≥4 ANC demonstrated significant associations with childhood undernutrition categories. However, in BDHS 2022, such associations were statistically significant for wasting, underweight, and multiple undernutrition. This indicates an improvement in the protective effect of maternal care against childhood undernutrition in Bangladesh, possibly resulting from the implementation of the nutrition-focused policies and strategies, such as the National Nutrition Policy 2015 and the Second National Plan of Action for Nutrition 2016–2025 [10,28]. A previous study identified an improvement in the quality of ANC, although access remained limited in rural area and among the low socioeconomic cohort and individuals with low access to information [29]. Additionally, the age range of children in the BDHS 2017–18 sample (6–35 months) was higher than that in the BDHS 2022 sample (6–23 months), which may partially explain why maternal utilization of CoC and receiving ≥4 ANC were not significantly associated with childhood undernutrition categories in the earlier survey. It is likely that the effects of maternal care from pregnancy to postpartum on children’s nutritional status abate as the children grow up.

The association between delivery assisted by SBA and childhood undernutrition categories was not statistically significant in either the BDHS 2017–18 or BDHS 2022 sample; however, delivery assisted by SBA was a significant protective factor against stunting, underweight, and any undernutrition in the pooled sample. Although not statistically significant in individual surveys, the consistent direction of the association across both BDHS 2017–18 and BDHS 2022 suggests a potentially stable association, and the pooled analysis increased the statistical precision, resulting in a statistically significant estimate [30]. Furthermore, this could also be influenced by differences in the distribution of time-varying covariates, e.g., education level of parents, wealth index, and maternal media exposure, across surveys [31]. Having delivery assisted by an SBA increases the likelihood of receiving a PNC within 48 hours of delivery. Therefore, the similarity in the direction and significance of associations between delivery assisted by SBA and receiving PNC within 48 hours with childhood undernutrition categories is not beyond assumption.

Consistent with the known pattern of childhood undernutrition in Bangladeshi, stunting (28.11%) was the most prevalent form, while only 8.33% of non-stunted children were either wasted or underweight or had a combination of both. This resulted in an overall 36.44% prevalence of any undernutrition in the analyzed sample. Accordingly, there was consistency in the direction and significance of association between maternal utilization of CoC and its components and both stunting and any undernutrition.

In neither individual surveys nor the pooled sample were maternal utilization of full CoC and ≥4 ANC significantly associated with lower odds of childhood stunting. This could be because childhood stunting results from chronic deprivation of essential macro- and micronutrients and is contributed to by multidimensional factors, e.g., poverty, poor hygiene and sanitation, and sub-optimum care. Research has shown that the decline in childhood stunting in Bangladesh was primarily driven by socioeconomic development, a decrease in poverty, maternal high educational attainment, and improved food security, rather than by nutrition-specific interventions delivered through maternal healthcare platforms [32,33].

In the pooled sample, delivery assisted by SBA and receiving PNC within 48 hours were significantly associated with low childhood stunting. In Bangladesh, delivery assisted by SBA, mostly associated with facility-based delivery, is consistently common among mothers from high socioeconomic groups, such as those with greater wealth and educational attainment—factors which are also strong predictors of childhood stunting [34]. Evidence has shown a significant difference in accessing institutional delivery among mothers from different socioeconomic groups, indicating the presence of pro-rich inequity in Bangladesh [35]. The analyzed dataset also shows a significantly high proportion of mothers from the richest families had delivery assisted by SBA (Supplementary Material Table S3). An explorative study revealed that poverty was the primary reason for mothers to rely on Traditional Birth Attendants (TBA) rather than SBA [36]. This scenario complies with the idea that a reduction in poverty and improvement of education level are also crucial to increase maternal healthcare utilization coverage and reduce childhood undernutrition subsequently [37].

In the analyzed sample, there were no survey-specific differences in the significance of effects of maternal utilization of full CoC and its components on childhood undernutrition categories. However, the stronger protective effects observed in the most recent survey (BDHS 2022) suggest improved quality of maternal care over the years. Sensitivity analyses conducted including children aged 0–2 years in both surveys also demonstrate similar pattern of associations, strengthening the validity of current findings. In sensitivity analyses, inclusion of children 0–2 years of age in the BDHS 2017–18 sample demonstrates stronger protective effects of maternal utilization of CoC on childhood undernutrition than those observed in analyses conducted with children 0–3 years of age in the same survey. This indicates that the beneficiary effects of maternal utilization of CoC on child nutrition tend to fade over time. There was no significant association of childhood wasting, underweight, and multiple forms of undernutrition with delivery assisted by SBA and receiving PNC within 48 hours. This could be due to considerable dropouts among the mothers from receiving full CoC. For example, in the wasting sample, 48% of mothers received ≥4 ANC, but only 34% of mothers both received ≥4 ANC and had delivery assisted by SBA (Supplementary Material Figure S1).

The coverage of receiving CoC and ≥4 ANC was lower in BDHS 2022 compared to BDHS 2017–18. This decline could be due to the COVID-19 pandemic, which substantially disrupted in-person maternal care services [38]. However, the pattern was the opposite for the coverage of having delivery assisted by SBA and PNC within 48 hours of delivery, primarily due to government policies and strategies emphasizing institutional delivery to reduce maternal and infant mortality.

### 4.1. Strengths and Limitations

To the best of our knowledge, this is the first study to observe the association between maternal utilization of CoC and childhood undernutrition using DHS program data from any country in the world. The use of BDHS 2017–18 and BDHS 2022 as data sources enhances the validity of results, as BDHS is amongst the most reliable data sources for public health research in Bangladesh. The inclusion of the latest two surveys allows for more robust analysis and an opportunity to compare survey-specific differences following the implementation of National Nutrition Policy 2015. We excluded <2% observations due to missingness in the covariates, indicating low selection bias.

In contrast, several limitations also warrant mentioning. As the surveys are cross-sectional, the causal association between maternal CoC and childhood undernutrition cannot be inferred. To estimate maternal utilization of CoC, the survey guidelines recommend considering live birth within three years preceding the survey for BDHS 2017–18 and within two years for BDHS 2022, respectively. Therefore, the analyzed sample included only children aged less than three years for BDHS 2017–18 and children aged less than two years for BDHS 2022, which limits the generalizability of the finding to children beyond this age range. In BDHS 2017–18 and BDHS 2022, mothers were interviewed for their utilization of CoC if they had a live birth three and two years preceding the surveys, respectively. Therefore, there remains the chances of information bias. Aspects such as quality of the maternal care and satisfaction level of mothers are important, especially while considering the long-term impact of CoC utilization. However, these are beyond the scope of this study. Furthermore, information related to some other potentially relevant covariates, including maternal dietary quality, household food security, sanitation quality, and infectious disease burden, was not available and not included in the analyses. These limitations should be taken into careful consideration while interpreting and extrapolating the findings.

### 4.2. Policy Implications and Future Scopes

The findings suggest that increasing the coverage of, and reducing dropout from, CoC for maternal health could further strengthen efforts to prevent childhood undernutrition, especially wasting and underweight, in Bangladesh. Existing evidence indicates there are challenges in strengthening nutrition services using MNCH platforms [39,40]. To translate the benefit of maternal utilization of CoC into the prevention of childhood stunting, delivery of quality nutrition care and a reduction in socioeconomic inequality should be prioritized. Understanding maternal capability to transform nutrition services into practice can also be an area of future research.

## 5. Conclusions

In Bangladesh, the effectiveness of maternal utilization of CoC in preventing childhood wasting, underweight, and multiple concurrent forms of undernutrition has improved over the last decade. However, a substantial gap was identified in translating the benefit of maternal utilization of CoC in reducing chronic childhood undernutrition, i.e., stunting. Improving the coverage and quality of maternal care, while minimizing the dropouts along the CoC and socioeconomic inequality, could result in more sustainable benefits in child nutrition outcomes in Bangladesh.

## Figures and Tables

**Table 1:** Distribution of childhood stunting, wasting, underweight, any undernutrition, and multiple undernutrition by maternal utilization of CoC, ≥4 ANC, delivery assisted by SBA, PNC within 48 hours of delivery, and maternal sociodemographic characteristics in the pooled sample.

Variables	Stunting (n = 6162)	Wasting (n = 6165)	Underweight (n = 6279)	Any Undernutrition (n = 6180)	Multiple Undernutrition (n = 6162)
Full CoC	[*p* < 0.001]	[*p* = 0.005]	[*p* < 0.001]	[*p* < 0.001]	[*p* < 0.001]
No	1300 (30.78)	401 (9.49)	913 (21.27)	1672 (39.49)	813 (19.25)
Yes	432 (22.28)	142 (7.32)	282 (14.20)	580 (29.80)	241 (12.43)
≥4 ANC	[*p* < 0.001]	[*p* = 0.088]	[*p* < 0.001]	[*p* < 0.001]	[*p* < 0.001]
No	1034 (31.43)	309 (9.38)	734 (21.92)	1323 (40.08)	652 (19.82)
Yes	698 (24.30)	234 (8.15)	461 (15.73)	929 (32.27)	402 (14.00)
Delivery by SBA	[*p* < 0.001]	[*p* = 0.023]	[*p* < 0.001]	[*p* < 0.001]	[*p* < 0.001]
No	904 (34.50)	256 (9.76)	628 (23.66)	1137 (43.31)	652 (21.45)
Yes	828 (23.38)	287 (8.10)	567 (15.64)	1115 (31.36)	492 (13.89)
PNC within 48 hours	[*p* < 0.001]	[*p* = 0.005]	[*p* < 0.001]	[*p* < 0.001]	[*p* < 0.001]
No	963 (33.20)	287 (9.88)	677 (23.03)	1222 (42.05)	608 (20.96)
Yes	769 (23.58)	256 (7.85)	518 (15.51)	1030 (31.46)	446 (13.68)
Mother’s age at delivery	[*p* = 0.004]	[*p* = 0.872]	[*p* = 0.054]	[*p* = 0.011]	[*p* = 0.177]
<19 years	310 (32.06)	88 (9.10)	201 (20.47)	386 (39.79)	177 (18.03)
19–30 years	1172 (26.95)	378 (8.69)	809 (18.27)	1537 (35.27)	719 (16.53)
31–49 years	250 (29.55)	77 (9.10)	185 (21.29)	329 (38.62)	158 (18.68)
Parity	[*p* < 0.001]	[*p* = 0.502]	[*p* < 0.001]	[*p* < 0.001]	[*p* < 0.001]
1	639 (27.26)	213 (9.09)	434 (18.14)	832 (35.42)	387 (16.51)
2–3	836 (26.77)	263 (8.42)	572 (17.98)	1107 (35.33)	499 (15.98)
>3	257 (36.98)	67 (9.61)	189 (26.81)	313 (44.84)	168 (24.17)
Mother’s education	[*p* < 0.001]	[*p* < 0.001]	[*p* < 0.001]	[*p* < 0.001]	[*p* < 0.001]
No education	139 (38.72)	52 (14.44)	127 (35.08)	180 (49.86)	166 (32.31)
Primary	559 (34.48)	153 (9.43)	392 (23.87)	698 (42.93)	347 (21.41)
Secondary	851 (28.10)	256 (8.45)	557 (18.03)	1104 (36.36)	489 (16.14)
Higher	183 (15.87)	82 (7.11)	119 (10.03)	270 (23.34)	102 (8.85)
Father’s education	[*p* < 0.001]	[*p* = 0.142]	[*p* < 0.001]	[*p* < 0.001]	[*p* < 0.001]
No education	317 (36.48)	92 (10.57)	242 (27.28)	404 (46.22)	208 (23.94)
Primary	691 (34.60)	174 (8.70)	465 (22.96)	848 (42.40)	423 (21.18)
Secondary	541 (26.01)	184 (8.85)	362 (17.16)	714 (34.23)	320 (15.38)
Higher	183 (15.05)	93 (7.65)	126 (10.02)	286 (23.44)	103 (8.47)
Mother’s occupation	[*p* < 0.001]	[*p* = 0.261]	[*p* = 0.362]	[*p* = 0.072]	[*p* = 0.131]
Not working	1063 (26.61)	364 (9.11)	762 (18.70)	1428 (35.63)	662 (16.57)
Working	669 (30.87)	179 (8.26)	433 (19.65)	824 (37.94)	392 (18.09)
Father’s occupation	[*p* = 0.236]	[*p* * = 0.660]	[*p* = 0.906]	[*p* = 0.340]	[*p* = 0.711]
Not working	14 (21.54)	4 (6.06)	12 (18.46)	20 (30.77)	10 (15.38)
Working	1718 (28.18)	539 (8.84)	1183 (19.04)	2232 (36.50)	1044 (17.12)
Problems in accessing healthcare	[*p* < 0.001]	[*p* = 0.526]	[*p* < 0.001]	[*p* < 0.001]	[*p* < 0.001]
Not a big problem	622 (24.36)	218 (8.54)	433 (16.56)	850 (33.14)	369 (14.45)
Big problem	1110 (30.76)	325 (9.00)	762 (20.80)	1402 (38.78)	685 (18.98)
BMI	[*p* < 0.001]	[*p* < 0.001]	[*p* < 0.001]	[*p* < 0.001]	[*p* < 0.001]
Underweight	353 (35.41)	140 (14.03)	300 (29.56)	474 (47.31)	268 (26.88)
Normal	1078 (28.57)	307 (8.13)	701 (18.27)	1379 (36.46)	620 (16.43)
Overweight	252 (22.40)	86 (7.64)	164 (14.27)	337 (29.88)	141 (12.53)
Obese	49 (18.35)	10 (3.75)	30 (10.79)	62 (23.13)	25 (9.36)
Wealth index	[*p* < 0.001]	[*p* = 0.089]	[*p* < 0.001]	[*p* < 0.001]	[*p* < 0.001]
Poorest	488 (36.89)	141 (10.65)	361 (27.0)	616 (46.42)	321 (24.26)
Poorer	410 (32.46)	113 (8.93)	268 (20.92)	506 (40.00)	240 (19.00)
Middle	335 (28.98)	94 (8.13)	225 (19.17)	417 (36.07)	208 (17.99)
Richer	297 (23.95)	102 (8.23)	193 (15.24)	404 (32.48)	163 (13.15)
Richest	202 (17.12)	93 (7.88)	148 (12.12)	309 (26.01)	122 (10.34)
Religion	[*p* = 0.271]	[*p* = 0.384]	[*p* = 0.975]	[*p* = 0.292]	[*p* = 0.798]
Muslim	1598 (28.30)	503 (8.90)	1095 (19.03)	2075 (36.63)	968 (17.14)
Others	134 (26.02)	40 (7.77)	100 (19.03)	177 (43.30)	86 (16.70)
Type of place of residence	[*p* < 0.001]	[*p* = 0.312]	[*p* = 0.019]	[*p* < 0.001]	[*p* = 0.043]
Urban	493 (24.23)	190 (9.33)	361 (17.38)	676 (33.10)	320 (15.72)
Rural	1239 (30.02)	353 (8.55)	834 (19.85)	1576 (38.09)	734 (17.79)
Division	[*p* < 0.001]	[*p* = 0.131]	[*p* < 0.001]	[*p* < 0.001]	[*p* < 0.001]
Barisal	189 (28.04)	63 (9.35)	119 (17.47)	243 (36.00)	111 (16.47)
Chittagong	287 (28.03)	87 (8.50)	182 (17.37)	369 (35.93)	162 (15.82)
Dhaka	194 (22.22)	79 (9.04)	131 (14.59)	271 (30.90)	114 (13.06)
Khulna	152 (23.35)	49 (7.52)	115 (17.50)	200 (30.58)	102 (15.67)
Mymensingh	259 (33.64)	77 (10.0)	183 (23.43)	331 (42.93)	160 (20.78)
Rajshahi	173 (27.24)	42 (6.61)	111 (16.84)	217 (33.91)	93 (14.65)
Rangpur	189 (26.40)	58 (8.10)	141 (19.42)	249 (34.45)	121 (16.90)
Sylhet	289 (35.29)	88 (10.73)	213 (25.69)	374 (45.67)	191 (23.32)
Survey round	[*p* < 0.001]	[*p* = 0.007]	[*p* = 0.084]	[*p* < 0.001]	[*p* = 0.037]
BDHS 2017–18	1385 (30.70)	371 (8.22)	900 (19.55)	1735 (38.33)	799 (17.71)
BDHS 2022	347 (21.03)	172 (10.41)	295 (17.61)	517 (31.26)	255 (15.45)
Overall sample	1732 (28.11)	543 (8.81)	1195 (19.03)	2252 (36.44)	1054 (17.10)

Note: ANC (Antenatal Care); BDHS (Bangladesh Demographic and Health Survey); CoC (Continuum of Care); BMI (Body Mass Index); PNC (Postnatal Care); SBA (Skilled Birth Attendants); * *p*-value calculated by Fisher’s exact test.

**Table 2:** Association between domains of maternal utilization of Continuum of Care and childhood undernutrition categories in BDHS 2017–18, BDHS 2022 and pooled sample.

Variables	BDHS 2017–18		BDHS 2022		Pooled Sample		*p*-Value ^‡^
	AOR * (95% CI)	*p*-Value	AOR * (95% CI)	*p*-Value	AOR ^†^ (95% CI)	*p*-Value	
Outcome: Stunting							
Full CoC							
No	Reference		Reference		Reference		
Yes	0.93 (0.78, 1.10)	0.39	0.90 (0.64, 1.28)	0.57	0.92 (0.78, 1.08)	0.29	0.62
≥4 ANC							
No	Reference		Reference		Reference		
Yes	0.94 (0.81, 1.10)	0.46	0.83 (0.61, 1.11)	0.21	0.92 (0.81, 1.06)	0.24	0.95
Delivery by SBA							
No	Reference		Reference		Reference		
Yes	0.86 (0.73, 1.01)	0.08	0.78 (0.57, 1.08)	0.13	0.84 (0.73, 0.97)	0.02	0.88
PNC							
No	Reference		Reference		Reference		
Yes	0.85 (0.72, 0.996)	0.04	0.79 (0.60, 1.05)	0.11	0.83 (0.72, 0.95)	0.01	0.95
Outcome: Wasting							
Full CoC							
No	Reference		Reference		Reference		
Yes	1.11 (0.81, 1.52)	0.53	0.49 (0.30, 0.80)	0.01	0.89 (0.68, 1.17)	0.41	0.25
≥4 ANC							
No	Reference		Reference		Reference		
Yes	1.22 (0.95, 1.57)	0.12	0.59 (0.39, 0.89)	0.01	1.00 (0.81, 1.24)	0.99	0.08
Delivery by SBA							
No	Reference		Reference		Reference		
Yes	0.97 (0.74, 1.28)	0.85	0.90 (0.57, 1.40)	0.64	0.94 (0.74, 1.19)	0.62	0.61
PNC							
No	Reference		Reference		Reference		
Yes	1.00 (0.76, 1.32)	1.00	0.78 (0.52, 1.16)	0.22	0.92 (0.73, 1.15)	0.46	0.89
Outcome: Underweight							
Full CoC							
No	Reference		Reference		Reference		
Yes	1.00 (0.81, 1.23)	0.96	0.65 (0.43, 0.98)	0.04	0.91 (0.75, 1.10)	0.32	0.25
≥4 ANC							
No	Reference		Reference		Reference		
Yes	1.01 (0.84, 1.21)	0.91	0.59 (0.42, 0.83)	0.003	0.90 (0.77, 1.06)	0.21	0.09
Delivery by SBA							
No	Reference		Reference		Reference		
Yes	0.85 (0.70, 1.02)	0.08	0.88 (0.62, 1.25)	0.48	0.85 (0.72, 0.998)	0.048	0.17
PNC							
No	Reference		Reference		Reference		
Yes	0.84 (0.70, 1.00)	0.05	0.87 (0.63, 1.21)	0.41	0.84 (0.72, 0.98)	0.03	0.14
Outcome: Any undernutrition							
Full CoC							
No	Reference		Reference		Reference		
Yes	0.95 (0.81, 1.13)	0.59	0.81 (0.59, 1.08)	0.15	0.91 (0.78, 1.06)	0.21	0.73
≥4 ANC							
No	Reference		Reference		Reference		
Yes	0.95 (0.82, 1.10)	0.50	0.81 (0.62, 1.06)	0.13	0.92 (0.81, 1.04)	0.20	0.88
Delivery by SBA							
No	Reference		Reference		Reference		
Yes	0.86 (0.74, 1.01)	0.06	0.77 (0.58, 1.02)	0.07	0.84 (0.73, 0.96)	0.01	0.58
PNC							
No	Reference		Reference		Reference		
Yes	0.85 (0.73, 0.99)	0.04	0.81 (0.63, 1.04)	0.10	0.83 (0.73, 0.94)	0.01	0.56
Outcome: Multiple undernutrition							
Full CoC							
No	Reference		Reference		Reference		
Yes	1.00 (0.81, 1.26)	0.96	0.52 (0.34, 0.81)	0.004	0.88 (0.72, 1.07)	0.19	0.15
≥4 ANC							
No	Reference		Reference		Reference		
Yes	1.05 (0.86, 1.28)	0.61	0.49 (0.35, 0.70)	<0.001	0.89 (0.76, 1.06)	0.19	0.06
Delivery by SBA							
No	Reference		Reference		Reference		
Yes	0.88 (0.72, 1.07)	0.19	0.81 (0.55, 1.18)	0.27	0.85 (0.72, 1.02)	0.08	0.53
PNC							
No	Reference		Reference		Reference		
Yes	0.86 (0.71, 1.05)	0.14	0.74 (0.53, 1.05)	0.10	0.83 (0.70, 0.98)	0.03	0.31

Note: * Adjusted for primary sampling units (clusters), strata, sample weight, mothers’ age at delivery, parity, mothers’ education, fathers’ education, mothers’ occupation, fathers’ occupation, problems in accessing healthcare (permission to go, accessing money, and distance to facility), BMI, wealth index, mothers’ religion, type of place of residence, and division of residence; ^†^ adjusted for primary sampling units (clusters), strata, sample weight, mothers’ age at delivery, parity, mothers’ education, fathers’ education, mothers’ occupation, fathers’ occupation, problem to access healthcare (permission to go, accessing money, and distance to facility), BMI, wealth index, mothers’ religion, type of place of residence, division of residence and survey round; ^‡^ *p*-values calculated by joint Wald tests; ANC (Antenatal Care); AOR (Adjusted Odds Ratio); BDHS (Bangladesh Demographic and Health Survey); CoC (Continuum of Care); PNC (Postnatal Care); SBA (Skilled Birth Attendant).

## Data Availability

The Bangladesh Demographic and Health Survey data are publicly available at https://dhsprogram.com/ (accessed on 9 June 2024). All the necessary information is provided either in the manuscript or in the Supplementary Material. Full statistical models and underlying datasets can be accessed from the corresponding author upon reasonable request.

## Supplementary Materials

The following supporting information can be downloaded at https://www.mdpi.com/article/10.3390/nu18121847/s1, Table S1: STROBE checklist; Table S2. Sensitivity analysis showing the association between domains of maternal utilization of Continuum of Care and childhood undernutrition categories among children 0–2 years of age in BDHS 2017–18, BDHS 2022 and pooled sample; Table S3: Distribution of mothers having delivery assisted by SBA by their household wealth index category; Figure S1: Distribution of the proportion of mothers receiving ≥4 ANC, ≥4 ANC and SBA-assisted delivery, and CoC across BDHS 2017–18, BDHS 2022, and pooled sample by childhood undernutrition categories.

## Abbreviations

The following abbreviations are used in this manuscript:

ANC	Antenatal Care
AOR	Adjusted Odds Ratio
BDHS	Bangladesh Demographic and Health Survey
BMI	Body Mass Index
CI	Confidence Interval
CoC	Continuum of Care
DHS	Demographic and Health Survey
MNCH	Maternal, Neonatal, and Child Health
PNC	Postnatal Care
SBA	Skilled Birth Attendant
SD	Standard Deviation
TBA	Traditional Birth Attendant
VIF	Variance Inflation Factor
