# Supplementary material for: Association Between Maternal Continuum of Care Utilization and Childhood Undernutrition in Bangladesh: Findings from Nationally Representative Surveys
Source: Nutrients. 2026 Jun 8;18(12):1847. doi: 10.3390/nu18121847 (PMC13305532; doi:10.3390/nu18121847)
Supplement: Supplementary file 1 [file nutrients-18-01847-s001.zip › nutrients-4327359-supplementary.pdf]

nutrients

MDPI

Supplementary materials

Association between maternal continuum of care utilization and childhood undernutrition in Bangladesh: Findings from nationally representative surveys

Contents

Table S1: STROBE checklist 1

Table S2. Sensitivity analysis showing the association between domains of maternal utilization of Continuum of Care and childhood undernutrition categories among children 0-2 years of age in BDHS 2017-18, BDHS 2022 and pooled sample 3

Table S3: Distribution of mothers having delivery assisted by SBA by their household wealth index category........ 5

Figure S1: Distribution of the proportion of mothers receiving ≥ 4 ANC, ≥ 4 ANC and SBA-assisted delivery, and CoC across BDHS 2017-18, BDHS 2022, and pooled sample by childhood undernutrition categories 6

Table S1: STROBE checklist

STROBE Statement—Checklist of items that should be included in reports of ***cross-sectional studies***

	Item No	Recommendation	Reporting location in the manuscript
**Title and abstract**	1	(*a*) Indicate the study's design with a commonly used term in the title or the abstract	Title
		(*b*) Provide in the abstract an informative and balanced summary of what was done and what was found	Abstract
**Introduction**			
Background/rationale	2	Explain the scientific background and rationale for the investigation being reported	Paragraph 3
Objectives	3	State specific objectives, including any prespecified hypotheses	Paragraph 3
**Methods**			
Study design	4	Present key elements of study design early in the paper	Paragraph 1 of study design, data sources and sample
Setting	5	Describe the setting, locations, and relevant dates, including periods of recruitment, exposure, follow-up, and data collection	Paragraph 1 of study design, data sources and sample
Participants	6	(*a*) Give the eligibility criteria, and the sources and methods of selection of participants	Paragraph 2 of study design, data sources and sample, Figure 1
Variables	7	Clearly define all outcomes, exposures, predictors, potential confounders, and effect modifiers. Give diagnostic criteria, if applicable	Exposure variables, Outcome

			variables, and Covariates
Data sources/ measurement	8*	For each variable of interest, give sources of data and details of methods of assessment (measurement). Describe comparability of assessment methods if there is more than one group	Statistical analysis
Bias	9	Describe any efforts to address potential sources of bias	Statistical analysis
Study size	10	Explain how the study size was arrived at	Paragraph 1, Figure 1
Quantitative variables	11	Explain how quantitative variables were handled in the analyses. If applicable, describe which groupings were chosen and why	Statistical analysis
Statistical methods	12	(*a*) Describe all statistical methods, including those used to control for confounding	Statistical analysis
		(*b*) Describe any methods used to examine subgroups and interactions	Statistical analysis
		(*c*) Explain how missing data were addressed	Figure 1, Statistical analysis
		(*d*) If applicable, describe analytical methods taking account of sampling strategy	Study design and sampling
		(*e*) Describe any sensitivity analyses	Statistical analysis
**Results**			
Participants	13*	(a) Report numbers of individuals at each stage of study—eg numbers potentially eligible, examined for eligibility, confirmed eligible, included in the study, completing follow-up, and analysed	Figure 1 and Paragraph 1 of Results
		(b) Give reasons for non-participation at each stage	Figure 1
		(c) Consider use of a flow diagram	Figure 1
Descriptive data	14*	(a) Give characteristics of study participants (eg demographic, clinical, social) and information on exposures and potential confounders	Table 1, paragraph 1 and 2 of result section
		(b) Indicate number of participants with missing data for each variable of interest	Figure 1
Outcome data	15*	Report numbers of outcome events or summary measures	Table 1
Main results	16	(*a*) Give unadjusted estimates and, if applicable, confounder-adjusted estimates and their precision (eg, 95% confidence interval). Make clear which confounders were adjusted for and why they were included	Table 2
		(*b*) Report category boundaries when continuous variables were categorized	Table 1
		(*c*) If relevant, consider translating estimates of relative risk into absolute risk for a meaningful time period	Not applicable
Other analyses	17	Report other analyses done—eg analyses of subgroups and interactions, and sensitivity analyses	Paragraph 6 of Results
**Discussion**			
Key results	18	Summarise key results with reference to study objectives	Paragraph 1 and 2
Limitations	19	Discuss limitations of the study, taking into account sources of potential bias or imprecision. Discuss both direction and magnitude of any potential bias	Strengths and limitations

Interpretation	20	Give a cautious overall interpretation of results considering objectives, limitations, multiplicity of analyses, results from similar studies, and other relevant evidence	Paragraph 2-6
Generalisability	21	Discuss the generalisability (external validity) of the study results	Paragraph 2-6
**Other information**			
Funding	22	Give the source of funding and the role of the funders for the present study and, if applicable, for the original study on which the present article is based	Funding statement

*Give information separately for exposed and unexposed groups.
Note: An Explanation and Elaboration article discusses each checklist item and gives methodological background and published examples of transparent reporting. The STROBE checklist is best used in conjunction with this article (freely available on the Web sites of PLoS Medicine at http://www.plosmedicine.org/, Annals of Internal Medicine at http://www.annals.org/, and Epidemiology at http://www.epidem.com/). Information on the STROBE Initiative is available at www.strobe-statement.org.

Table S2. Sensitivity analysis showing the association between domains of maternal utilization of Continuum of Care and childhood undernutrition categories among children 0-2 years of age in BDHS 2017-18, BDHS 2022 and pooled sample

Variables	BDHS 2017-18 AOR* (95% CI)	p-value	BDHS 2022 AOR* (95% CI)	p-value	Pooled sample AOR† (95% CI)	p-value	p-value‡
**Outcome: Stunting**							
**Full CoC**							
No	Reference		Reference		Reference		
Yes	0..88 (0.70, 1.09)	0.25	0.90 (0.64, 1.28)	0.57	0.89 (0.74, 1.08)	0.23	0.54
**≥ 4 ANC**							
No	Reference		Reference		Reference		
Yes	0.89 (0.74, 1.07)	0.21	0.83 (0.61, 1.11)	0.21	0.88 (0.75, 1.03)	0.11	0.85
**Delivery by SBA**							
No	Reference		Reference		Reference		
Yes	0.86 (0.70, 1.05)	0.13	0.78 (0.57, 1.08)	0.13	0.83 (0.70, 0.99)	0.03	0.99
**PNC**							
No	Reference		Reference		Reference		
Yes	0.83 (0.68, 1.00)	0.06	0.79 (0.60, 1.05)	0.11	0.81 (0.69, 0.96)	0.01	0.97
**Outcome: Wasting**							
**Full CoC**							
No	Reference		Reference		Reference		
Yes	1.13 (0.78, 1.66)	0.51	0.49 (0.30, 0.80)	0.01	0.84 (0.62, 1.13)	0.25	0.31
**≥ 4 ANC**							
No	Reference		Reference		Reference		
Yes	1.17 (0.86, 1.60)	0.32	0.59 (0.39, 0.89)	0.01	0.91 (0.71, 1.16)	0.44	0.19
**Delivery by SBA**							
No	Reference		Reference		Reference		
Yes	0.99 (0.72, 1.36)	0.94	0.90 (0.57, 1.40)	0.64	0.95 (0.73, 1.23)	0.70	0.38
**PNC**							
No	Reference		Reference		Reference		
Yes	0.99 (0.72, 1.36)	0.95	0.78 (0.52, 1.16)	0.22	0.90 (0.70, 1.15)	0.40	0.55
**Outcome: Underweight**							

Variables	BDHS 2017-18 AOR* (95% CI)	p-value	BDHS 2022 AOR* (95% CI)	p-value	Pooled sample AOR† (95% CI)	p-value	p-value‡
**Full CoC**							
No	Reference		Reference		Reference		
Yes	0.94 (0.71, 1.24)	0.67	0.65 (0.43, 0.98)	0.04	0.84 (0.67, 1.06)	0.15	0.25
**≥ 4 ANC**							
No	Reference		Reference		Reference		
Yes	0.91 (0.71, 1.17)	0.47	0.59 (0.42, 0.83)	0.003	0.80 (0.66, 0.98)	0.03	0.049
**Delivery by SBA**							
No	Reference		Reference		Reference		
Yes	0.82 (0.66, 1.04)	0.10	0.88 (0.62, 1.25)	0.48	0.83 (0.69, 1.01)	0.06	0.18
**PNC**							
No	Reference		Reference		Reference		
Yes	0.80 (0.63, 1.00)	0.053	0.87 (0.63, 1.21)	0.41	0.82 (0.68, 0.99)	0.04	0.16
**Outcome: Any undernutrition**							
**Full CoC**							
No	Reference		Reference		Reference		
Yes	0.87 (0.71, 1.06)	0.17	0.81 (0.59, 1.08)	0.15	0.84 (0.71, 0.10)	0.046	0.54
**≥ 4 ANC**							
No	Reference		Reference		Reference		
Yes	0.87 (0.73, 1.04)	0.13	0.81 (0.62, 1.06)	0.13	0.86 (0.74, 0.99)	0.04	0.63
**Delivery by SBA**							
No	Reference		Reference		Reference		
Yes	0.84 (0.70, 1.01)	0.06	0.77 (0.58, 1.02)	0.07	0.81 (0.69, 0.94)	0.01	0.62
**PNC**							
No	Reference		Reference		Reference		
Yes	0.81 (0.68, 0.98)	0.03	0.81 (0.63, 1.04)	0.10	0.80 (0.69, 0.93)	0.004	0.48
**Outcome: Multiple undernutrition**							
**Full CoC**							
No	Reference		Reference		Reference		
Yes	0.97 (0.72, 1.29)	0.81	0.52 (0.34, 0.81)	0.004	0.81 (0.63, 1.03)	0.09	0.31
**≥ 4 ANC**							
No	Reference		Reference		Reference		
Yes	0.93 (0.71, 1.21)	0.60	0.49 (0.35, 0.70)	<0.001	0.77 (0.62, 0.95)	0.013	0.04
**Delivery by SBA**							
No	Reference		Reference		Reference		
Yes	0.86 (0.67, 1.09)	0.21	0.81 (0.55, 1.18)	0.27	0.84 (0.68, 1.02)	0.08	0.78
**PNC**							
No	Reference		Reference		Reference		
Yes	0.83 (0.65, 1.05)	0.13	0.74 (0.53, 1.05)	0.10	0.80 (0.65, 0.98)	0.03	0.62

Note: *Adjusted for primary sampling units (clusters), strata, sample weight, mothers' age at delivery, parity, mothers' education, fathers' education, mothers' occupation, fathers' occupation, problem to access healthcare (permission to go, accessing money, and distance to facility), BMI, wealth index, mothers' religion, type of place of residence, and division of residence; †Adjusted for primary sampling units (clusters), strata, sample weight, mothers' age at delivery, parity, mothers' education, fathers' education, mothers' occupation, fathers' occupation, problem to access healthcare (permission to go, accessing money, and distance to facility), BMI, wealth index, mothers' religion, type of place of residence, division of residence and survey round; ‡p-values calculated by joint Wald tests; ANC (Antenatal Care); AOR (Adjusted Odds Ratio); BDHS (Bangladesh Demographic and Health Survey); CoC (Continuum of Care); PNC (Postnatal Care); SBA (Skilled Birth Attendant)

Table S3: Distribution of mothers having delivery assisted by SBA by their household wealth index category

Household categories by wealth index	Delivery assisted by SBA		Total
	No	Yes	
Poorest (n)	901	422	1,323
%	68.1	31.9	100
Poorer (n)	692	571	1,263
%	54.79	45.21	100
Middle (n)	468	688	1,156
%	40.48	59.52	100
Richer (n)	389	851	1,240
%	31.37	68.63	100
Richest (n)	170	1,010	1,180
%	14.41	85.59	100
Total (n)	2,620	3,542	6,162
%	42.52	57.48	100

Overall P-value < 0.001

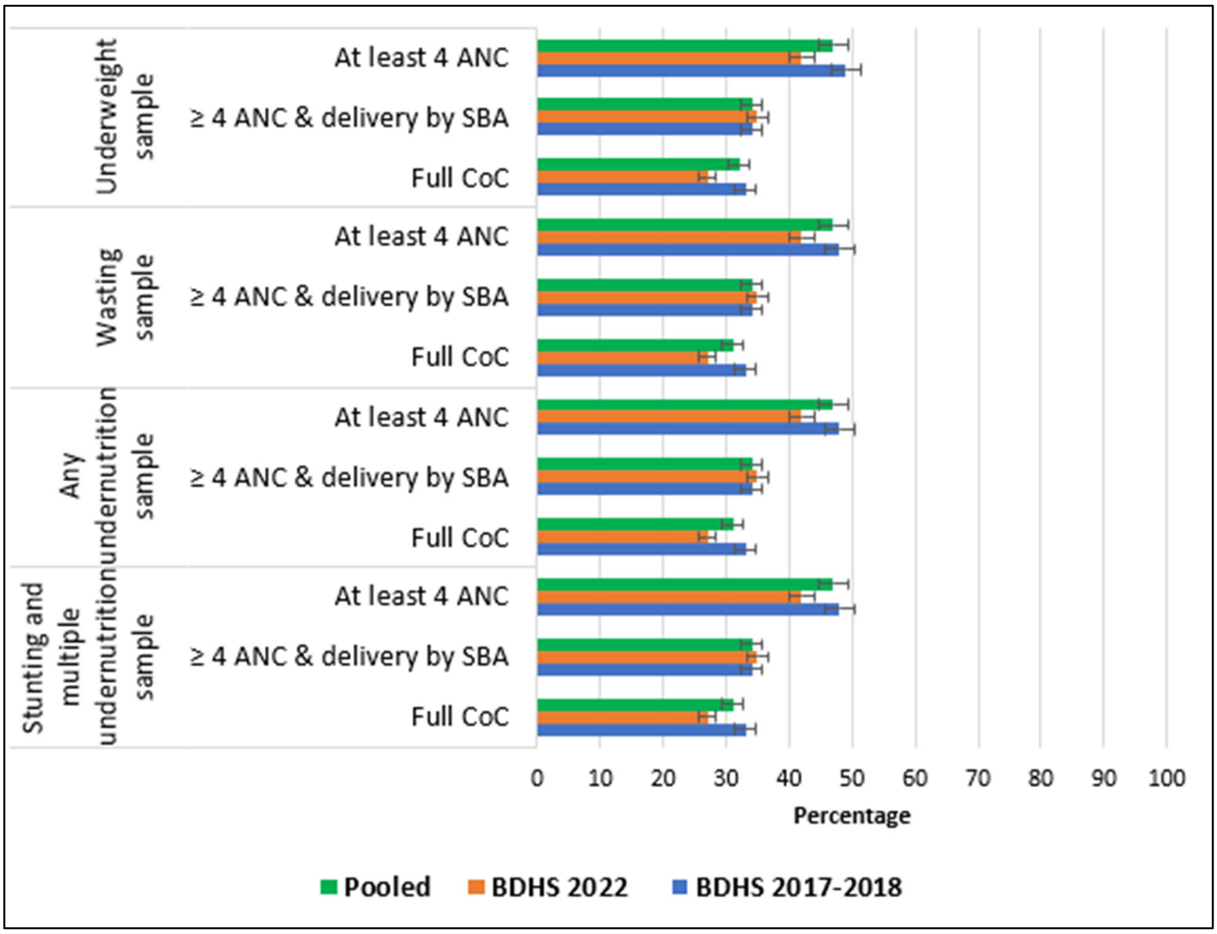

Figure S1: Distribution of the proportion of mothers receiving ≥ 4 ANC, ≥ 4 ANC and SBA-assisted delivery, and CoC across BDHS 2017-18, BDHS 2022, and pooled sample by childhood undernutrition categories
